# First Simultaneous Lidar Observations of Thermosphere‐Ionosphere Fe and Na (TIFe and TINa) Layers at McMurdo (77.84°S, 166.67°E), Antarctica With Concurrent Measurements of Aurora Activity, Enhanced Ionization Layers, and Converging Electric Field

**DOI:** 10.1029/2020GL090181

**Published:** 2020-10-19

**Authors:** Xinzhao Chu, Yukitoshi Nishimura, Zhonghua Xu, Zhibin Yu, John M. C. Plane, Chester S. Gardner, Yasunobu Ogawa

**Affiliations:** ^1^ Cooperative Institute of Research in Environmental Sciences and Department of Aerospace Engineering Sciences University of Colorado Boulder Boulder CO USA; ^2^ Department of Electrical and Computer Engineering and Center for Space Physics Boston University Boston MA USA; ^3^ Bradley Department of Electrical and Computer Engineering Virginia Polytechnic Institute and State University Blacksburg VA USA; ^4^ Harbin Institute of Technology Shenzhen China; ^5^ School of Chemistry University of Leeds Leeds UK; ^6^ Department of Electrical and Computer Engineering University of Illinois Urbana IL USA; ^7^ National Institute of Polar Research Tokyo Japan

**Keywords:** Antarctica, lidar observations, thermosphere‐ionosphere Fe layers, thermosphere‐ionosphere Na layers, aurora, enhanced ionization

## Abstract

We report the first simultaneous, common‐volume lidar observations of thermosphere‐ionosphere Fe (TIFe) and Na (TINa) layers in Antarctica. We also report the observational discovery of nearly one‐to‐one correspondence between TIFe and aurora activity, enhanced ionization layers, and converging electric fields. Distinctive TIFe layers have a peak density of ~384 cm^−3^ and the TIFe mixing ratio peaks around 123 km, ~5 times the mesospheric layer maximum. All evidence shows that Fe^+^ ion‐neutralization is the major formation mechanism of TIFe layers. The TINa mixing ratio often exhibits a broad peak at TIFe altitudes, providing evidence for in situ production via Na^+^ neutralization. However, the tenuous TINa layers persist long beyond TIFe disappearance and reveal gravity wave perturbations, suggesting a dynamic background of neutral Na, but not Fe, above 110 km. The striking differences between distinct TIFe and diffuse TINa suggest differential transport between Fe and Na, possibly due to mass separation.

## Key Points

- First simultaneous lidar observations show striking differences between distinct TIFe and diffuse TINa, suggesting differential transport
- We report the observational discovery of the correlation of TIFe with aurora and enhanced ionization, revealing plasma‐neutral coupling
- All observational evidence points to the Fe^+^ ion‐neutralization origin of TIFe while suggesting a background of neutral Na above 110 km

## 1 Introduction

The metallic atoms and ions in the Earth's upper atmosphere are of extraterrestrial origin as they are produced by the ablation and sputtering of cosmic dust (Plane, 2012). The metal atoms and ions undergo complex chemical reactions and dynamical transport over multiple temporal and spatial scales and form various metal layers in both ionized and neutral states (e.g., Bishop & Earle, 2003; Chu, Yu, et al., 2011; Mathews, 1998; Plane, 2003). The meteoric metal layers are of great interest scientifically for several reasons. They are excellent tracers for profiling temperatures and winds along with various waves in the mesosphere and thermosphere; they are a natural laboratory for exploring upper atmospheric composition, chemistry, dynamics, energetics, and electrodynamics; and they provide information on cosmic dust input flux, entry velocity, and composition in the terrestrial and other planetary atmospheres. Observation and modeling of thermosphere‐ionosphere metal (designated TIMt) layers, discovered in recent years, have opened a new door to advance understanding of fundamental processes in the space‐atmosphere interaction region (Chu & Yu, 2017; Plane et al., 2015), especially in the E–F regions where measurements of the neutral atmosphere are scarce but plasma‐neutral interactions are rich.

However, the detection of TIMt layers with lidars is very challenging, because their low densities and narrow absorption linewidths require very high detection sensitivity and frequency accuracy. The permanent (main) layers of metal atoms (75–105 km) have been observed from the ground for nearly a century (e.g., Bernard, 1938; Bowman et al., 1969; Chu & Papen, 2005; Hunten, 1967; Slipher, 1929), but neutral metal layers in the thermosphere were not discovered from ground‐based observations until Chu, Yu, et al. (2011) reported the first lidar observations of TIFe layers from Antarctica. Since then, TIMt observations have been reported from high to low latitudes, including TIFe (Chu et al., 2013, 2016; Lübken et al., 2011), TINa (Gao et al., 2015; Liu et al., 2016; Tsuda et al., 2015; Wang et al., 2012), and thermospheric K layers (TIK) (Friedman et al., 2013). The only known simultaneous observations of two TIMt species were made at Arecibo (18.35°N), showing similar behaviors of TINa and TIK (Raizada et al., 2015).

Here we report the first simultaneous, common‐volume lidar observations of TIFe and TINa neutral layers, made with Fe Boltzmann and Na Doppler lidars, over McMurdo (77.84°S, 166.67°E), showing striking differences. Such observations of multiple TIMt species help differentiate their possible sources. Chu, Yu, et al. (2011) proposed that the TIFe layers are produced through neutralization of converged Fe^+^ layers via direct recombination with electrons. Chu and Yu (2017) developed a TIFe model based on this hypothesis which successfully replicated the lidar observations on 28 May 2011. Nevertheless, this theory does not rule out other formation mechanisms, for example, direct meteoric deposition via sputtering, and direct transport of neutral metals from their main layers. Due to different volatility, mass, chemistry, and elemental abundance in the cosmic dust (e.g., Jessberger et al., 2001), the source injection rate, chemical production/loss, and transport of Fe and Na will differ substantially. Therefore, the various potential sources of TIMt will lead to different [TIFe]/[TINa] ratios. The concurrent Fe/Na lidar, aurora, ionosonde, and Defense Meteorological Satellite Program (DMSP) observations described here provide an exceptional opportunity to advance TIMt science and reveal complex atmosphere–ionosphere‐magnetosphere coupling processes.

## 2 Observations With Collocated Fe Boltzmann and Na Doppler Lidars

The University of Colorado installed an Fe Boltzmann lidar at Arrival Heights Observatory near McMurdo in December 2010 (Chu, Huang, et al., 2011; Chu, Yu, et al., 2011) and added a Na Doppler lidar in January 2018, with support from the United States Antarctic Program and Antarctica New Zealand. By probing two absorption lines, the Fe lidar detects neutral Fe atoms at 372 and 374 nm, and temperatures are derived from the two‐channel ratios using the Boltzmann technique (Chu et al., 2002; Gelbwachs, 1994). The Na lidar employs a narrowband laser transmitter tuned to the D_2a_ line at 589 nm, and a three‐frequency Doppler‐ratio technique to measure Na density, temperature, and vertical wind simultaneously (Chu & Papen, 2005) with high‐efficiency receiver architecture (Smith & Chu, 2015).

**Figure 1:**
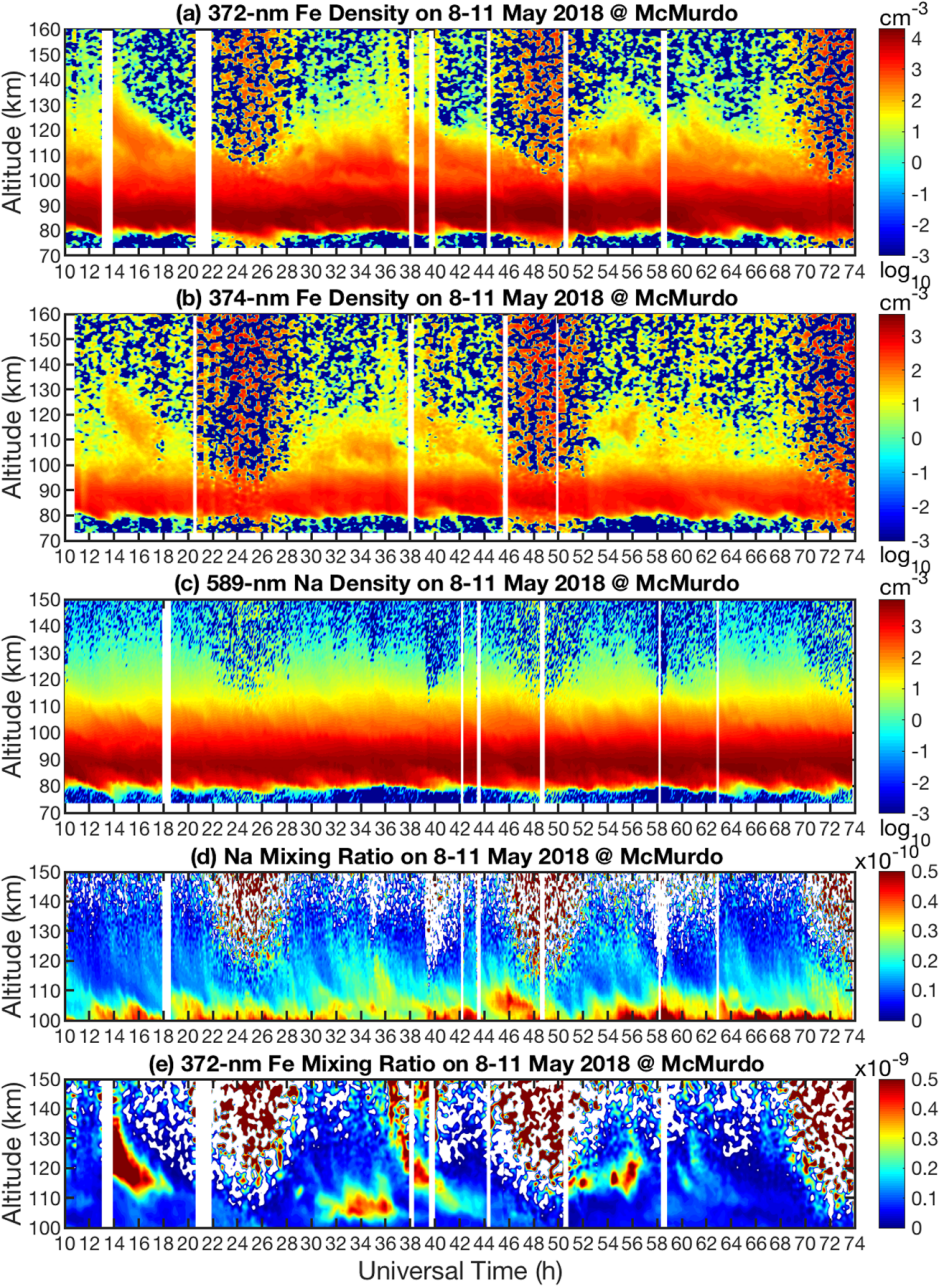
Simultaneous, common‐volume lidar observations of neutral Fe and Na layers on 8–11 May 2018 at McMurdo. (a) 372‐nm and (b) 374‐nm Fe densities, (c) Na densities, (d) Na mixing ratio, and (e) 372‐nm Fe mixing ratio. Raw photon count data have resolutions of 48 m and 1 min for Fe lidar and 24 m and 9 s for Na lidar. The Fe densities are retrieved with resolutions of 0.25 h and 0.5 km and oversampled to 0.1 h and 0.1 km. The Na density is retrieved and sampled at 0.1 h and 0.96 km.

Illustrated in Figures 1a and 1b are the neutral Fe layers observed in the 372‐ and 374‐nm channels on 8–11 May 2018, while Figure 1c shows the simultaneous observations of neutral Na layers at 589 nm. Daytime data (22–28, 46–52, and 70–74 UT) have insufficient signal‐to‐noise ratios for TIFe/TINa detection; therefore, we focus on nighttime observations. The main metal layers distributed from ~75 to ~100 km exhibit highly similar density variations over the entire measurement period, and the Na layer peaks (~90 km) above the Fe peak (~85 km) as expected. The tenuous and persistent Na and Fe in the lower thermosphere (100–110 km) are largely due to upward diffusion from the main layers and a chemical steady state between the atoms and their corresponding ions, where ionization via charge transfer with ambient NO^+^ and O_2_^+^ is balanced by formation of molecular ions followed by dissociative recombination with electrons (Plane, 2003). However, the stark contrast between the TIFe and TINa layers above 110 km is observed for the first time. The high‐altitude TIFe layers are distinctive, dynamical, and “detached” from the main layer, but the associated TINa layers are diffuse. Various TIFe patterns occur during three nights. Around 13 UT on 8 May, a TIFe layer forms and starts to descend from ~145 km and merges with the main layer near 110 km around 17 UT. This TIFe layer has an extraordinarily high density (~384 cm^−3^ at 123 km) and shows up clearly in both Fe channels. The 9 May night exhibits several downward‐phase TIFe layers after 28 UT with increasing layer heights until ~36.6 UT when a TIFe layer descends from ~160 km with initially fast phase speeds. The phase speed slows below 130 km and merges with the main layer around 42 UT. On 10 May, a TIFe layer is visible from 54–58 UT with the maximum height at ~140 km. Two TIFe layers occur from 59–62 UT with the downward phase progression of a gravity wave period ~1.5 h. The most prominent TIFe layers appear at ~13–15, 36–38, and 60–62 UT, suggesting a diurnal cycle. Yu et al. (2020) discussed the potential dynamical drivers of TIFe diurnal variations including electric field and tidal winds. This complex topic is beyond the scope of the current work but will be explored in future work.

The Na and Fe volume mixing ratios (Figures 1d and 1e) are calculated by dividing the Na and 372‐nm Fe density profiles by the corresponding total atmospheric number densities at McMurdo provided by the MSISE00 model (Picone et al., 2002). Figure 1d clearly exhibits the TINa layer features of downward phase progression of gravity waves with periods ranging from ~24 min to many hours. These TINa layers have low ratios of the wave crest to trough, similar to wave perturbations in the neutral atmosphere but in contrast to several high‐density layers that dominate the Fe mixing ratio. A weak TINa layer descends from ~145 km around 14 UT in Figure 1d corresponding to the strongest TIFe event in Figure 1e, and the TINa around 56 UT descending from 140 km matches the TIFe layer descending from 140 km at the same time. Several converged TINa layers occur after 16 UT above 125 km, after the TIFe has disappeared.

**Figure 2:**
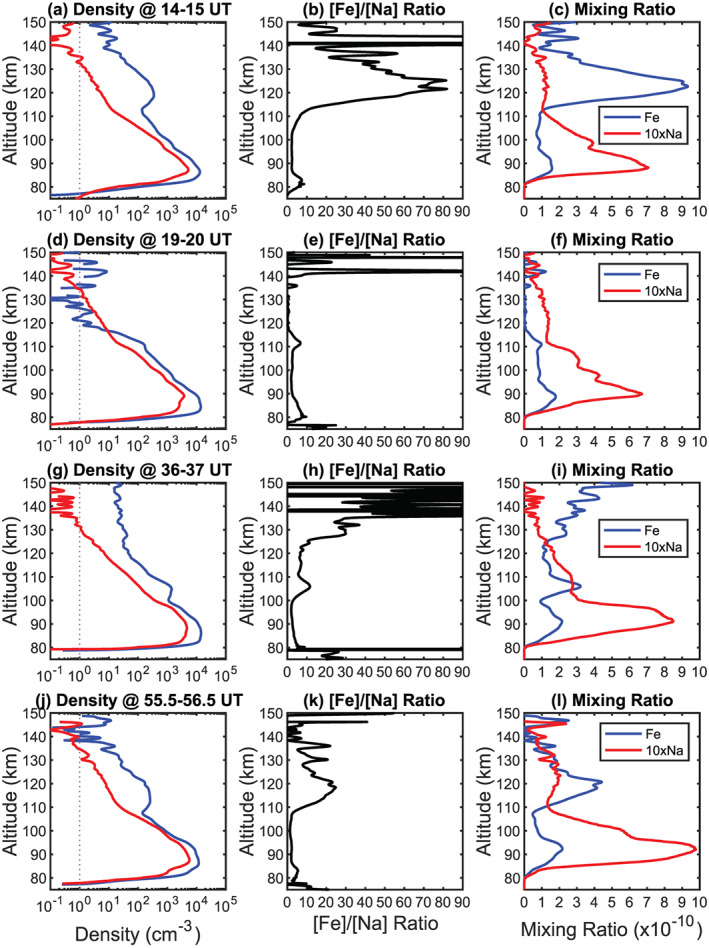
Hourly vertical profiles of Fe (blue) and Na (red) densities (first column), Fe/Na density ratios (second column), and Fe and Na mixing ratios (third column) for 14–15, 19–20, 36–37, and 55.5–56.5 UT on 8–11 May 2018 at McMurdo. Na mixing ratio is multiplied by a factor of 10 for illustration. For the nighttime hourly profiles, the Na and Fe detection limits at altitudes of 120–150 km range from ~0.2–1 and ~2–10 cm^−3^, respectively. The uncertainty (precision) of Na density varies from ~0.1% at the mesospheric peak to ~2%–10% in the TIMt. The uncertainty (precision) of Fe density changes from the main peak ~1% to 20%–30% above 120 km.

Four hourly Fe and Na density profiles and density ratio profiles are plotted in Figure 2 for comparison. At 14–15 UT, the [Fe]/[Na] density ratios vary dramatically, from ~2 around 90–100 km to ~90 around 120–125 km (Figure 2b). However, at 19–20 UT, the TIFe density above 117 km drops below the TINa density (Figures 2d–2f), confirming the disappearance of the TIFe in Figure 1. The Fe and Na mixing ratio profiles plotted in the third column of Figure 2 highlight these striking differences. The mixing ratios of the main Fe layer peak around 85 km (~2 × 10^−10^) and then decrease with altitude until ~105 km. In contrast, the TIFe mixing ratios increase dramatically to ~9.3 × 10^−10^ around 122.6 km (Figure 2c). This 400%–500% increase of the Fe mixing ratio indicates significant production of neutral Fe atoms in the thermosphere. The TINa mixing ratios remain smaller than the mesospheric layer peak around 90 km (6–10 × 10^−11^). The Na and Fe density profiles (Figures 2a and 2j) exhibit similar slopes (scale heights) from ~90 to ~110 km and then a turning point at ~110 km where the TIMt emerge. Correspondingly, the Na and Fe mixing ratios have steep negative gradients from 90 to ~110 km, facilitating the upward diffusion to ~110 km. Above 110 km, the Na mixing ratio exhibits a weak, broad peak at the altitudes of dramatically increased TIFe mixing ratios (Figures 2c and 2l), providing strong evidence for in situ production of Na. Figures 2g–2i present a different case where the TINa mixing ratio continuously decreases above 110 km but the TIFe mixing ratio increases above 120 km and the TIFe density reaches ~20 cm^−3^ at 150 km.

## 3 Concurrent Observations of Aurora, Ionization Layers, and Converging Electric Field

**Figure 3:**
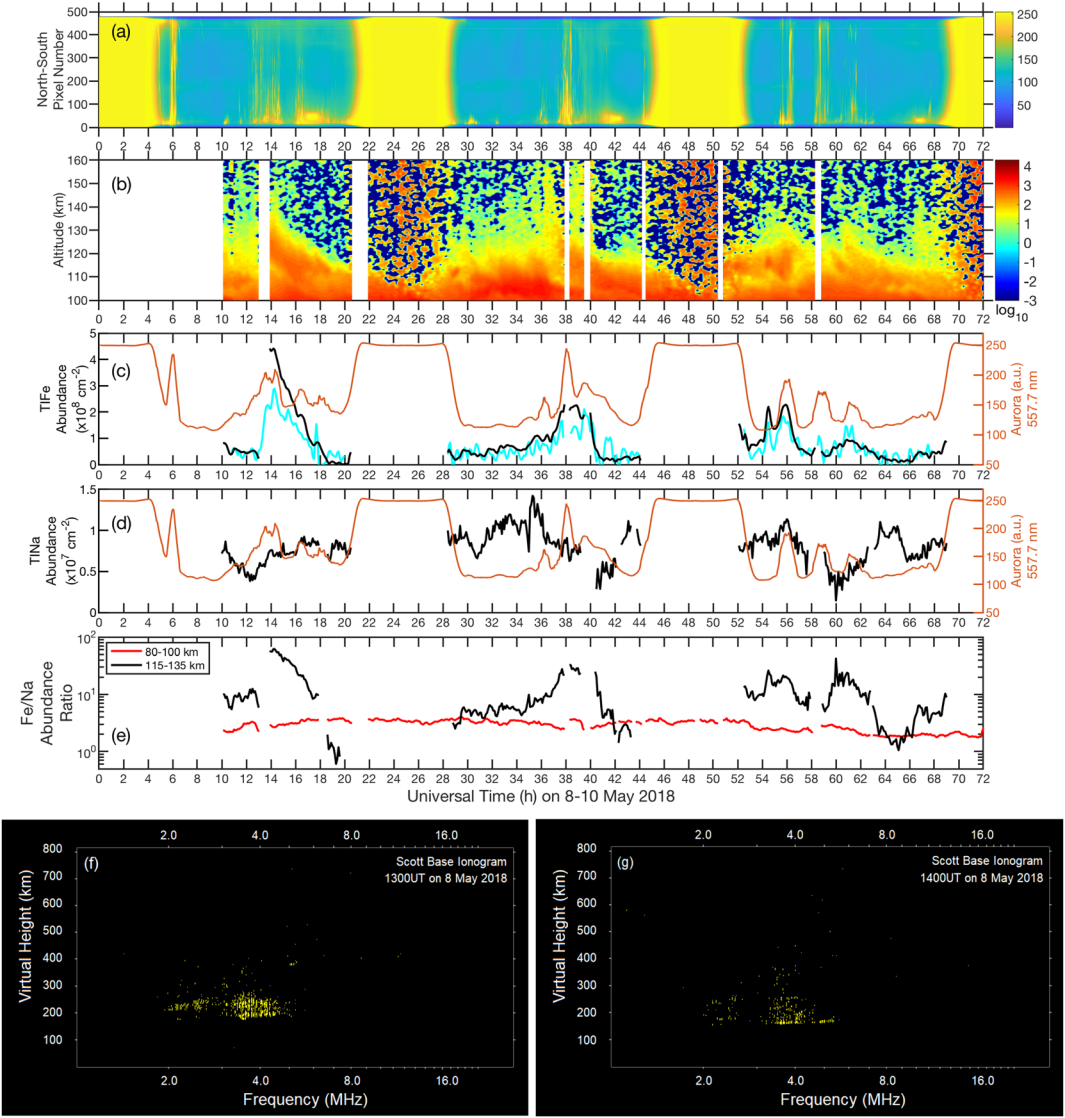
Concurrent measurements on 8–10 May 2018 at McMurdo/Scott Base: (a) Aurora 557.7 nm image, (b) 372‐nm TIFe layers, (c) TIFe column abundance (110–140 km) in 372 (black) and 374 nm (cyan) vs. aurora intensity (red), (d) TINa column abundance (115–135 km, black) vs. aurora intensity (red), (e) Fe and Na column abundance ratios in the main layer (red) and in the TIMt (black), (f) and (g) Scott Base ionograms at 1300 and 1400 UT on 8 May 2018. The aurora keogram is plotted against the north‐south pixel number at a time resolution of 2 min, where the south is to the top. The bright emission at 0–5, 20–29, 44–53, and after 68 UT is the daylight. The spot around pixel 30 at 17, 42, and 67 UT is the moonlight. Aurora intensity in (c) and (d) is Hamming smoothed with a 30‐min window. The 374‐nm TIFe abundance is multiplied by a factor of 4 for illustration. Daytime TIFe and TINa abundances have been excluded.

TIFe layer modeling (Chu & Yu, 2017) has shown that neutralization of converged Fe^+^ ion layers is a major source of TIFe layers via Fe^+^‐electron recombination, and auroral particle precipitation accelerates Fe^+^ neutralization via enhancing electron density in the E–F regions. Several known TIFe events coincided with solar/geomagnetic storms (Chu et al., 2016; Chu, Yu, et al., 2011). The lidar measurements reported here were made in the recovery phase of a geomagnetic storm occurred on 5 May 2018 (http://wdc.kugi.kyoto‐u.ac.jp/dst_realtime/201805/index.html and http://wdc.kugi.kyoto‐u.ac.jp/ae_realtime/201805/index_20180508.html). Therefore, we investigate concurrent observations of aurora and ionization. A collocated auroral all‐sky camera at Arrival Heights (Ogawa et al., 2020) observed the auroral activity using the oxygen green line at 557.7 nm every 4 s. A keogram of the auroral image in the north‐south direction is plotted versus time in Figure 3a. Almost every distinct TIFe layer above ~115 km (Figure 3b) corresponds to an auroral event, which is reflected in enhanced green line emissions. To examine this correlation further, the auroral intensity at 1° north of zenith at McMurdo (around pixel 80) is coplotted with the column abundance of the TIFe layer (115–140 km) in Figure 3c. Both the 372‐ and 374‐nm TIFe abundances correlate closely with the auroral intensity. Between 12 and 20 UT, not only does the strongest TIFe layer correspond well to the stronger aurora at 13–14 UT, but the two smaller TIFe peaks correspond to the two weaker auroral events at 16 and 18 UT. The TIFe events between 32 and 42 UT also exhibit good correspondence with auroral activity during the same period, although lidar data gaps at 38 and near 40 UT miss the TIFe peaks at the time of the auroral peaks. TIFe events from 54–64 UT match two of the auroral peaks, but a lidar data gap (due to detuned lasers) from 58–59 UT missed the middle auroral peak. A small peak in the TIFe column abundance at ~54.5 UT does not correspond to any auroral event. However, Figure 1 shows that this small peak results from sporadic Fe layers below 120 km (Plane, 2003), not a TIFe layer descending from above.

The TIFe modeling (Chu & Yu, 2017) demonstrates that significantly enhanced Fe^+^ densities are required to yield sufficient Fe production rates through recombination, in order to match the observed TIFe densities. The Scott Base ionosonde located within several kilometers from Arrival Heights detected enhanced ionization layers around and below 200 km (virtual) at the times of strong TIFe layers (Figures 3f and 3g). The event on 8 May exhibited an enhanced ionization layer at ~190 km (virtual) at 13:00 UT and then a concentrated layer around 160 km (virtual) with the maximum frequency reaching 5.5 MHz at 14:00 UT. The strongest TIFe layer emerged between 14 and 15 UT, but the ionosonde recorded data hourly on the hour, so we do not know whether a higher electron density occurred during this TIFe event. Enhanced ionization layers also occurred on 9 and 10 May below 200 km, corresponding to the lidar‐observed TIFe events (see Figure S2 in the supporting information). The critical frequency of 5.5 MHz corresponds to an electron density [*e*^−^] of ~3.7 × 10^5^ cm^−3^, much higher than the normal nighttime electron density (~5 × 10^3^ cm^−3^). Auroras can enhance electron and ion densities; however, with [*e*^−^] ~3.7 × 10^5^ cm^−3^, the e‐folding lifetime of NO^+^ and O_2_^+^ is very short, ~9–10 s, due to rapid dissociative recombination with electrons (loss rate: *k*[*e*^−^] ≈ 3 × 10^7^cm^3^*s*^−1^ × 3.7 × 10^5^cm^−3^ = 0.11 s^−1^) (Chu, Yu, et al., 2011; Plane, 2003). The polar cap auroras which occurred during these observations were weak, thus unlikely to produce ionization fast enough to compensate for this loss rate. Therefore, it is very likely that the enhanced ionization layers mainly consist of metallic ions. Fe^+^ is usually the dominant species (Kopp, 1997), supporting the TIFe modeling prediction.

Achieving enhanced Fe^+^ density requires mechanisms to converge metal ions vertically and/or horizontally. Besides the vertical convergence by vertical shears of vertical and horizontal winds under high‐inclination polar magnetic field and by polar electric field in certain orientations (Chu & Yu, 2017), the aurora‐induced converging electric field may help converge Fe^+^ ions horizontally. Such a converging electric field occurred in the DMSP‐measured plasma drift velocity over McMurdo during the lidar observations (see Figure S1). At 11:50 and 13:31 UT, the DMSP shows large shears of the velocity along with enhanced electron precipitation and field‐aligned current (FAC) at −80° geomagnetic latitude (McMurdo). The positive slope of the sunward velocity along the satellite track corresponds to the converging north‐south electric field, and tens of mV/m converging electric field occurred near −80° MLAT. Such converging electric field and enhanced FAC are consistent with the enhanced ionization layers discussed above.

While TIFe observations correspond well with aurora and enhanced ionization, the TINa layers do not exhibit such obvious correlations as demonstrated in Figure 3d where the TINa column abundance (115–135 km) is plotted against the aurora intensity. The ratios of [Fe]/[Na] column abundances in the altitude ranges of 80–100 and 115–135 km are compared in Figure 3f. The main layer [Fe]/[Na] ratios are ~3, but the [TIFe]/[TINa] ratios vary significantly from ~60 at ~14 UT to less than 1 at ~19 UT.

## 4 Discussion

The significant increase of the TIFe mixing ratio (≥500%) above 110 km demonstrates that there must be significant production of neutral Fe in the thermosphere, because pure vertical transport of neutral Fe from the main deposition region cannot substantially increase its mixing ratio (Cox et al., 1993). The nearly one‐to‐one correspondence between the TIFe layers and auroral pulses suggests that sputtering of meteoroids is not the source of these high‐altitude TIFe layers since there is no reason for meteoroid sputtering to correlate with auroral activity. Aurora sputtering of metals from meteoric smoke particles during energetic particle precipitation events (von Zahn et al., 1987) can also be ruled out because smoke particles have negligible concentrations above 100 km due to their substantial mass (Hunten et al., 1980; Plane et al., 2015). The meteoric Chemical Ablation Model (CABMOD) also shows negligible injection rates of Fe above ~120 km by sputtering (Carrillo‐Sánchez et al., 2015, 2016). Therefore, both the direct upward transport (advection and diffusion) of neutral Fe from its permanent layer and the direct neutral Fe deposition via sputtering are ruled out. The observational evidence presented here clearly points to the Fe^+^ ion neutralization origin of TIFe layers. The enhanced ionization layers observed by the ionosonde and close correlation with auroral activity support the TIFe modeling results (Chu & Yu, 2017).

Auroral intensity smoothed with a 30‐min Hamming window correlates well with the TIFe layers (Figure 3). This result indicates that the TIFe response to auroral activity is not instantaneous, but occurs over a longer time scale of ~30 min, which is comparable to the time scale of Fe^+^ neutralization simulated in Chu and Yu (2017). Such correlation suggests that TIFe and aurora are likely driven by some common factors, for example, the disturbed polar electric field and enhanced electron precipitation. Once the background convection electric field transports Fe^+^ from the main deposition region to the E–F regions, the converging electric field accumulates Fe^+^ ions horizontally and neutral wind shears converge Fe^+^ vertically to achieve the required high Fe^+^ density (e.g., 10^5^–10^6^ cm^−3^), as evidenced by the enhanced ionization seen in the ionogram around 150 km. Recombination of Fe^+^ with electrons produces TIFe layers with a rate proportional to [Fe^+^][e^−^], and the process can be accelerated by the enhanced [Fe^+^] and electron densities associated with auroral activity (Chu & Yu, 2017).

The different behavior of the TINa layers is striking. Na^+^ ions should experience the same transport processes as Fe^+^, forming converged Na^+^ layers. Neutralization of converged Na^+^ will then lead to neutral Na production, and this mechanism has been invoked to explain TINa observations elsewhere (Gao et al., 2015; Tsuda et al., 2015). These earlier observations show that the densities of TINa layers range from 1 to 6.3 cm^−3^ at 130–170 km (Gao et al., 2015), much smaller than the McMurdo TIFe, but the ratios of the TINa density to the background (contrast) are quite high, similar to the McMurdo TIFe. The low‐contrast and long‐lasting TINa layers observed at McMurdo suggest a more complex picture. Na^+^ direct‐recombination rate is ~1/3 of Fe^+^ (1.7 × 10^−12^ vs. 5.1 × 10^−12^ cm^3^ s^−1^ at 500 K) (Nahar et al., 1997; Verner & Ferland, 1996). The average Fe:Na ratio from meteoric ablation was recently estimated to be 4.3 (Carrillo‐Sánchez et al., 2020). If the Fe^+^:Na^+^ above 125 km has a ratio close to this, then the Na production rate would be ~13 times smaller than that of Fe (note that above 125 km direct recombination is the dominant route to produce the neutral atoms in TIMt, Plane et al., 2015). When the Fe^+^ neutralization is fast enough to overcome the Fe loss (e.g., Fe^+^ production by charge transfer and aurora‐induced ionization) and produce distinctive TIFe layers (Chu & Yu, 2017), the Na^+^ neutralization may barely exceed the Na loss, resulting in weak TINa layers at the same altitude range of strong TIFe layers (Figure 2c). In fact, TINa may experience faster loss than TIFe, due to reionization of Na caused by converged, dominant meteoric ions such as Fe^+^, Mg^+^, and Si^+^, as the ionization potential of Na is so much lower than these other metals and charge transfer of Na with these ions could be as fast as the Langevin limit. The lower production rate and the higher loss of TINa likely cause the density of TINa to be much lower than TIFe when both layers occur at the same time (Figure 2a).

Given that Arecibo observations show similar behaviors of TINa and TIK, despite their mass difference (Raizada et al., 2015), it is surprising that the tenuous TINa persists after the distinctive TIFe disappears (e.g., Figures 2d–2f) which seems to happen frequently. Comparing the Fe and Na mixing ratios in Figures 1d and 1e, there is Na, but not Fe, above 110 km at 17, 34, 43, and 64 UT. Some TINa layers show similar broad peaks as in Figure 2c, but some exhibit continuous mixing ratio decrease above 110 km (Figure 2i). Such low‐contrast but long‐lasting TINa as well as the clear gravity wave perturbations with periods as short as ~20 min suggest a dynamic background of neutral Na, but not Fe, above 110 km that is modulated by various waves propagating upward into the thermosphere. Such striking differences suggest differential transport between Fe and Na atoms and/or ions, because aforementioned differences in chemical production and loss between TINa and TIFe should enhance the Fe background compared to Na. For the cases of a continuous mixing ratio decrease as in Figure 2i, diffusive and advective transport of neutral Na can contribute to TINa above 110 km; but for cases with mixing ratios slightly increasing above 110 km (Figures 2c, 2f, and 2l), direct transport of neutrals from the main layer at McMurdo will not work due to the mixing ratio constraint (Cox et al., 1993), although this may still be possible for Na^+^.

Aurora‐related Joule/particle heating happens often between 100 and 200 km in the auroral zone (Cole, 1962) and may penetrate below 110 km to the altitudes with relatively high Na mixing ratios. The heating‐induced upwelling motion can transport light species, such as neutral Na, to the thermosphere above 110 km, but is probably less effective on heavy Fe atoms. Patches of neutral Na with elevated mixing ratios could therefore form above 110 km in the auroral zone and then be transported over McMurdo located at the poleward edge of the auroral oval. Without converged metal ions (Fe^+^, Mg^+^) nearby, ionization of Na atoms by charge transfer with ambient NO^+^ and O_2_^+^ will be relatively slow, and such loss could be compensated by advective transport as described above due to frequent aurora events, forming the dynamic background of Na in the polar region. Roddy et al. (2004) observed a Mg^+^ (with a mass number very close to Na^+^) layer peaking ~0.5 km higher than Fe^+^ around 118 km. The possible mass separation of Fe and Na atoms and ions during transport should be explored through more correlative measurements and numerical modeling.

## 5 Conclusions

Observational discoveries reported here, enabled by the first simultaneous lidar measurements of TIFe and TINa in Antarctica, establish the close correlation between TIMt and magnetospheric‐ionospheric processes for the first time and reveal striking and unexpected differences between distinct TIFe and diffuse TINa. TIFe layers emerge as very dynamical, highly distinctive, and detached phenomena from the permanent Fe layers and are correlated with aurora events and enhanced ionization layers on nearly one‐to‐one correspondence. The dramatically increased TIFe mixing ratios (~5 times the mesospheric layer maximum), high TIFe densities (~10–400 cm^−3^), and close correlation with enhanced ionization and aurora strongly support the theory that neutralization of Fe^+^ ions via direct recombination with electrons is the major production mechanism of TIFe layers (Chu & Yu, 2017). The TINa mixing ratio often exhibits a broad peak at TIFe altitudes, providing strong evidence for in situ production via Na^+^ neutralization. However, the tenuous TINa layers persist long beyond TIFe disappearance and reveal clear downward phase progression of gravity wave perturbations with periods ranging from tens of minutes to many hours. There is likely a dynamic background of neutral Na, but not Fe, above 110 km at McMurdo. Differential transport between Fe and Na atoms and/or ions is suggested, likely due to the mass separation of heavy Fe and light Na. We imagine that Joule/particle heating in the auroral zone serves as a “fountain” to frequently “launch” Na‐rich air parcels into the thermosphere by heating‐induced upwelling winds. Horizontal transport of these air parcels by neutral winds could then form a dynamic background of Na above 110 km. Quantitative investigation of the TIMt formation mechanisms and connections with aurora, ionization, converging electric field, Joule/particle heating, and neutral winds is beyond the scope of this work, but will undoubtedly inspire future modeling and observational work.

## Supporting information

Supporting Information S1

## Data Availability

DMSP data were obtained through cedar.openmadrigal.org. The data shown in this work can be downloaded online (from https://data.mendeley.com/datasets/3ykf8xz8kc/2).
