# Supplementary material for: First Simultaneous Lidar Observations of Thermosphere‐Ionosphere Fe and Na (TIFe and TINa) Layers at McMurdo (77.84°S, 166.67°E), Antarctica With Concurrent Measurements of Aurora Activity, Enhanced Ionization Layers, and Converging Electric Field
Source: Geophys Res Lett. 2020 Oct 19;47(20):e2020GL090181. doi: 10.1029/2020GL090181 (PMC7685115; doi:10.1029/2020GL090181)
Supplement: Supplementary file 1 — Supporting Information S1 [file GRL-47-e2020GL090181-s001.pdf]

Geophysical Research Letters

Supporting Information for

First Simultaneous Lidar Observations of Thermosphere-Ionosphere Fe and Na (TIFe and TINa) Layers at McMurdo (77.84°S, 166.67°E), Antarctica with Concurrent Measurements of Aurora Activity, Enhanced Ionization Layers, and Converging Electric Field

Xinzhao Chu[1]*, Yukitoshi Nishimura[2], Zhonghua Xu[3], Zhibin Yu[4], John M. C. Plane[5], Chester S. Gardner[6], and Yasunobu Ogawa[7]

[1]Cooperative Institute of Research in Environmental Sciences & Department of Aerospace Engineering Sciences, University of Colorado Boulder, USA

[2]Department of Electrical and Computer Engineering and Center for Space Physics, Boston University, Boston, Massachusetts, USA

[3]Bradley Department of Electrical and Computer Engineering, Virginia Polytechnic Institute and State University, Blacksburg, VA, USA

[4]Harbin Institute of Technology, Shenzhen, China

[5]School of Chemistry, University of Leeds, Leeds, LS2 9JT, United Kingdom

[6]Department of Electrical and Computer Engineering, University of Illinois, USA

[7] National Institute of Polar Research, Tokyo, Japan

*Corresponding Author: Xinzhao.Chu@Colorado.edu

Contents of this file

Introduction
Figures S1 to S3
Table S1
Plate S1

Introduction

There were Defense Meteorological Satellite Program (DMSP) spacecraft passes over McMurdo during the lidar measurements, and there were also concurrent ionosonde observations at Scott Base. These data support the thermosphere-ionosphere Fe and Na (TIFe and TINa) sciences discussed in the main article. This supporting information document provides the plots of DMSP data and Scott Base ionograms in Figures S1 and S2, respectively. Figure S3 provides the contour plots of Fe/Na density ratio, Fe and Na mixing ratios in the full altitude range of 75–150 km observed by collocated Fe Boltzmann and Na Doppler lidars on 8–11 May 2018 at Arrival Heights Observatory near McMurdo, Antarctica. Furthermore, Table S1 lists the parameters for the Fe and Na lidars, and Plate S1 shows a map of DMSP footprints near McMurdo Station.

DMSP data were obtained through cedar.openmadrigal.org. Scott Base ionosonde data are available from http://www.sws.bom.gov.au/World_Data_Centre/1/1. The ionogram data were downloaded from
ftp://ftp-out.sws.bom.gov.au/wdc/wdc_ion_archive/sct4d/cln/2018/scdn20180508.zip
ftp://ftp-out.sws.bom.gov.au/wdc/wdc_ion_archive/sct4d/cln/2018/scdn20180509.zip
ftp://ftp-out.sws.bom.gov.au/wdc/wdc_ion_archive/sct4d/cln/2018/scdn20180510.zip.
The ionogram viewer website is http://www.sws.bom.gov.au/HF_Systems/1/3. Note that the ionosonde data were available only at the minute 00 of each hour for 8–11 May 2018.

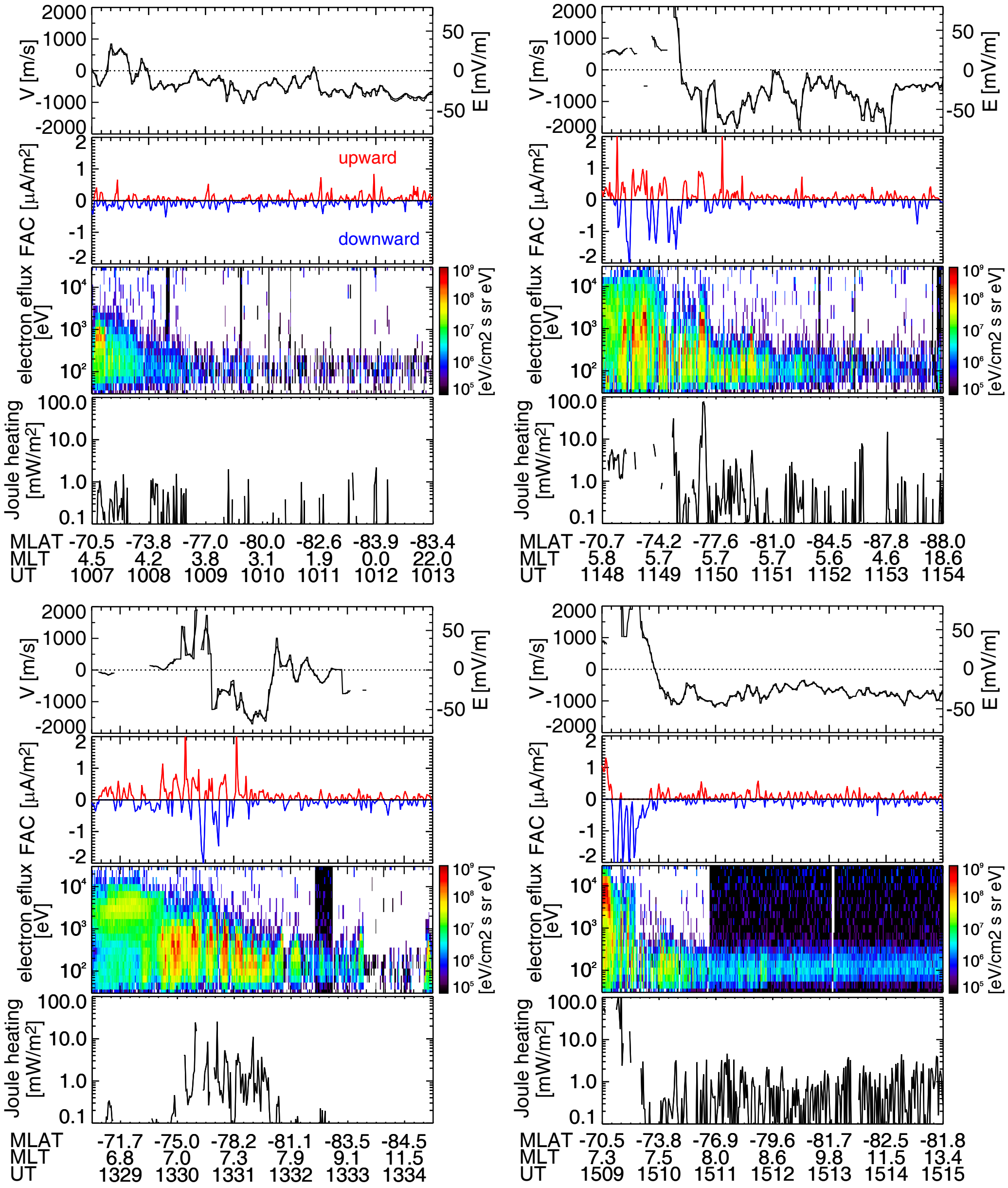

Figure S1. Four consecutive DMSP passes in the southern hemisphere over McMurdo on 8 May 2018. The panels shown are the cross-track horizontal velocity (positive sunward), field-aligned current, electron precipitating energy flux, and Joule heating. The along-track electric field (positive equatorward) was obtained as the cross product of the cross-track velocity and magnetic field. The field-aligned current was obtained from the magnetometer assuming stationary magnetic field perturbations. The Joule heating is obtained from the Pedersen conductance inferred from the electron flux (Robinson formula) and the along-track electric field. Because the cross-track electric field is unknown, this is a minimum estimate of the Joule heating.

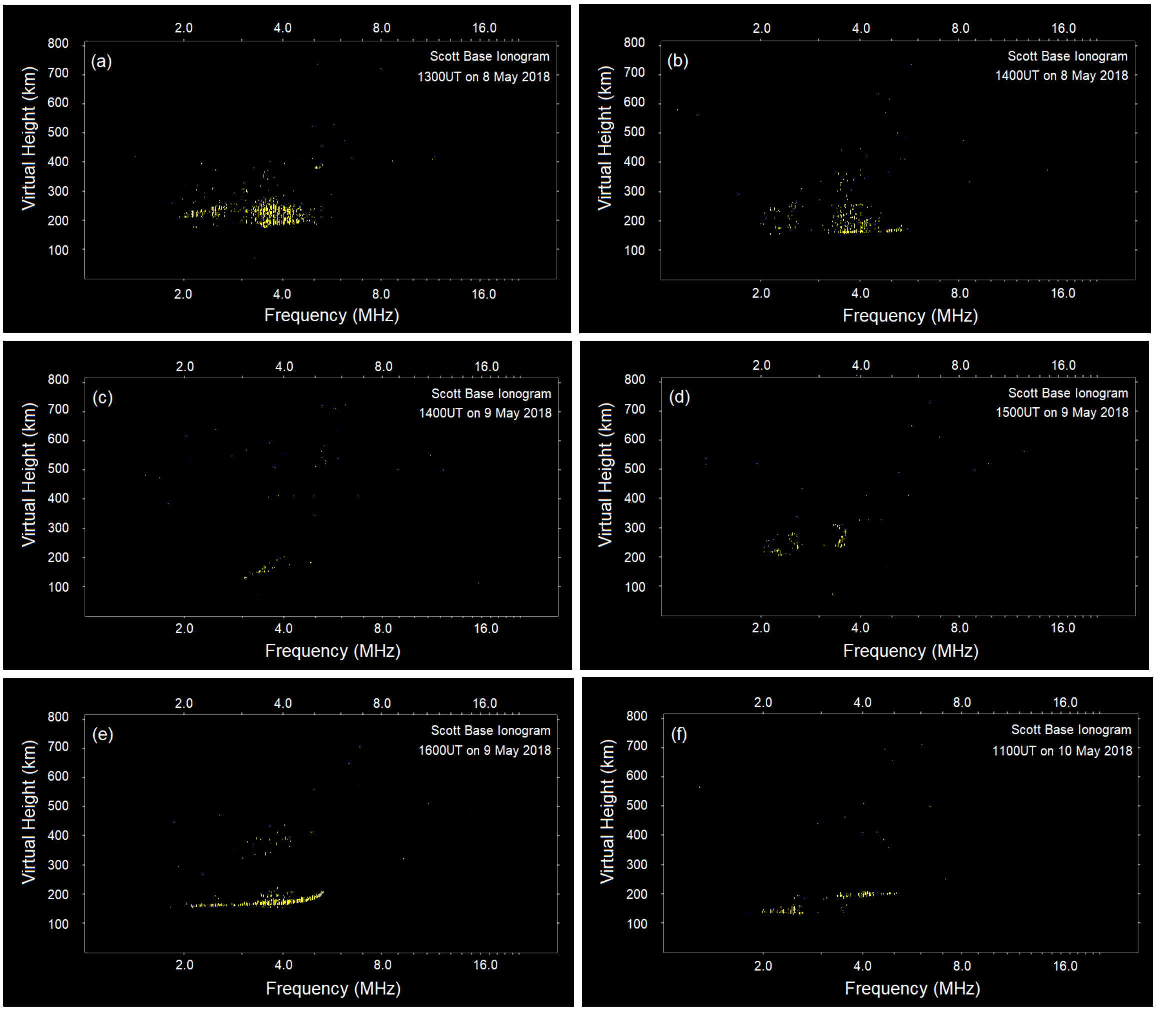

Figure S2. Ionograms of Scott Base ionosonde showing enhanced ionization layers in the E and lower F regions during the TIFe layer occurrence on 8–10 May 2011. (a) and (b) Scott Base ionograms at 1300UT and 1400UT on 8 May 2018. (c), (d), and (e) Scott Base ionograms at 1400UT, 1500UT, and 1600UT on 9 May 2018. (f) Scott Base ionogram at 1100UT on 10 May 2018.

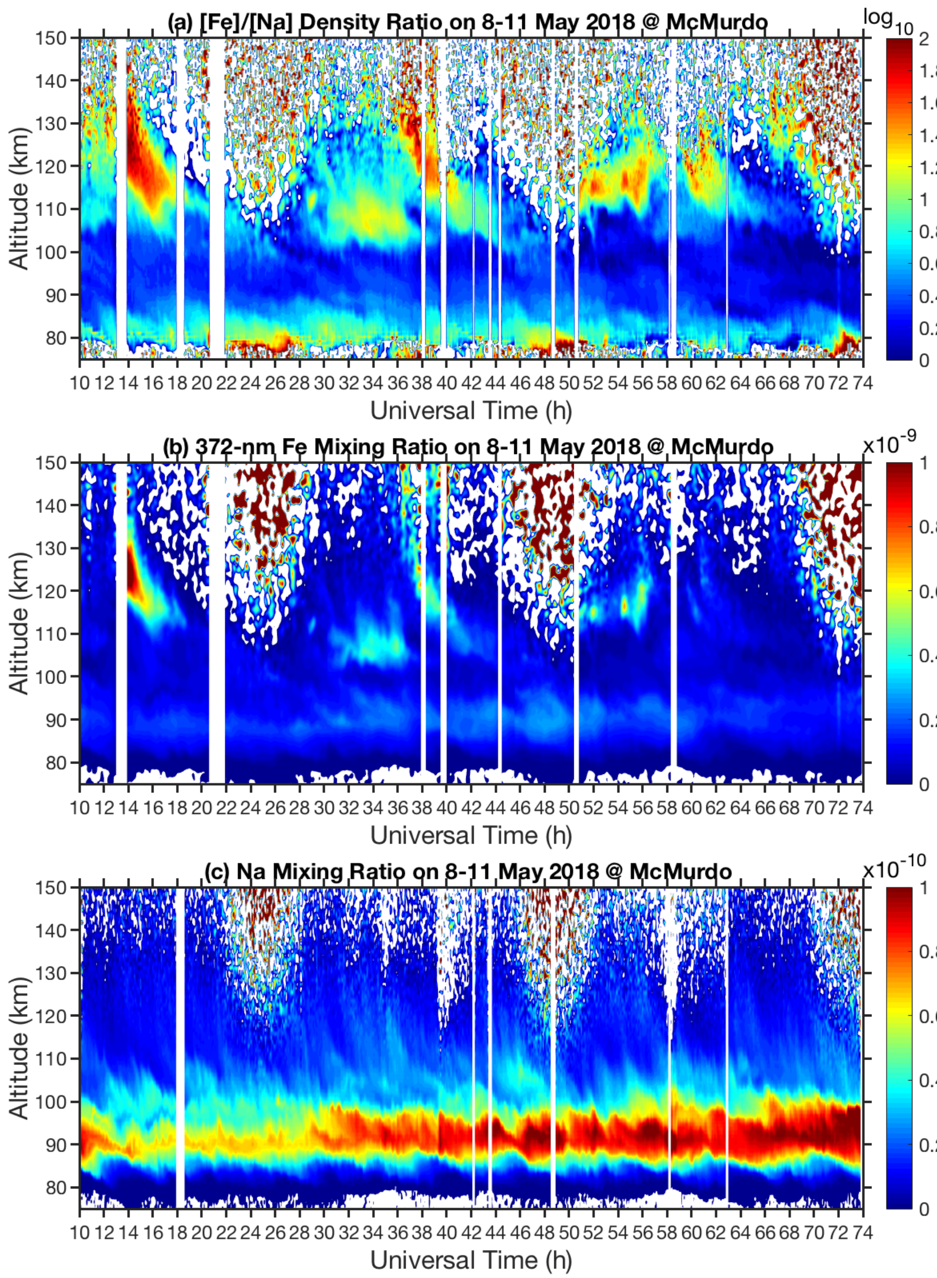

Figure S3. (a) Fe/Na density ratio, (b) 372-nm Fe mixing ratio, and (c) Na mixing ratio in the full altitude range (75–150 km) measured by the collocated Fe Boltzmann and Na Doppler lidars on 8–11 May 2018 at Arrival Heights Observatory (77.84ºS, 166.67ºE) near McMurdo, Antarctica. The Fe and Na densities, density ratios, and mixing ratios were derived at resolutions of 0.25 h and 0.96 km with oversampling display resolutions of 0.1 h and 0.1 km.

Table S1.

Parameters of Fe Boltzmann and Na Doppler Lidars in Nighttime Configuration

Parameters	Fe Boltzmann Lidar (372 nm)	Fe Boltzmann Lidar (374 nm)	STAR Na Doppler Lidar (589 nm)
Laser Pulse Energy (mJ)	60–90	60–90	15–30
Pulse Repetition Rate (Hz)	33.2	34.1	50
Laser Power (W)	2–3	2–3	0.75–1.5
Seed Laser Frequency Accuracy (MHz)	±54	±54	±1
Pulsed Laser Linewidth RMS (MHz)	572	409	100
Pulsed Width FWHM (ns)	60–100	60–100	7–10
Number of Frequency	1	1	3
AOM Frequency Shift (MHz)	N/A	N/A	±750
Telescope Diameter (cm)	40	40	81
Interference Filter Bandwidth FWHM (nm)	4.2	4.2	1
PMT Quantum Efficiency	28%	28%	40%
Original Temporal Resolution (s)	60	60	4.5
Original Range Resolution (m)	48	48	24

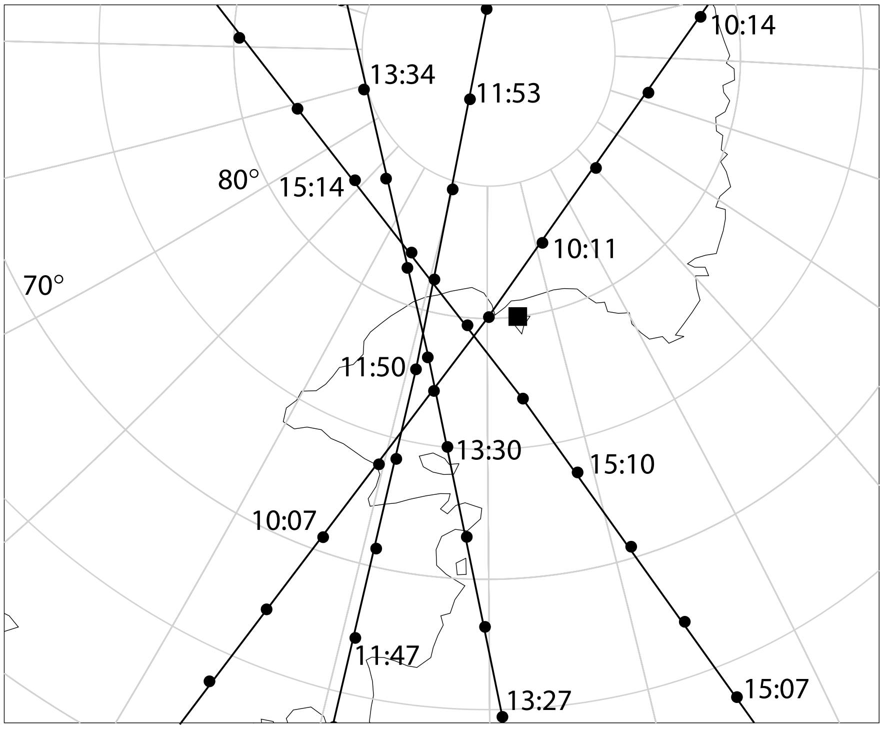

Plate S1. An Antarctic map showing the footprints of four consecutive DMSP passes on 8 May 2018 over McMurdo as discussed in Figure S1. The black square represents the location of McMurdo, and the map is labeled in magnetic latitudes and universal time.
